# Hantavirus Infection with Renal Failure and Proteinuria, Colorado, USA, 2019

**DOI:** 10.3201/eid2602.191349

**Published:** 2020-02

**Authors:** Swati Chand, Sangharsha Thapa, Shelley Kon, Steven C. Johnson, Eric M. Poeschla, Carlos Franco-Paredes, Alfonso J. Rodríguez-Morales, Salim Mattar, Andrés F. Henao-Martínez

**Affiliations:** Kathmandu University School of Medical Sciences, Kathmandu, Nepal (S. Chand, S. Thapa);; University of Colorado School of Medicine, Aurora, Colorado, USA (S. Kon, S.C. Johnson, E.M. Poeschla, C. Franco-Paredes, A.F. Henao-Martínez);; Hospital Infantil de México Federico Gómez, Mexico City, Mexico (C. Franco-Paredes);; Universidad Tecnológica de Pereira, Pereira, Colombia (A.J. Rodríguez-Morales);; Universidad de Córdoba, Montería, Colombia (S. Mattar)

**Keywords:** hantavirus, Seoul virus, viruses, acute kidney injury, proteinuria, Colorado, North America, United States

## Abstract

In North America, hantaviruses commonly cause hantavirus pulmonary syndrome (HPS). Clinical descriptions of hantavirus-associated renal disease in the Americas are scarce. Herein, we discuss the case of a 61-year-old man whose predominant manifestations were acute kidney injury and proteinuria. Clinical recognition of renal signs in hantavirus infections can reduce risk for death.

In the United States, 20–40 hantavirus cases are reported annually. Human infections result from inhalation of aerosolized secretions of infected rodents. Hantavirus pulmonary syndrome (HPS) is associated with pneumonitis and has a broad clinical spectrum that ranges from mild or no symptoms to fulminant respiratory failure. Hemorrhagic fever with renal syndrome (HFRS) is a characteristic clinical entity that manifests with fever, hypotension, and renal failure (*1*) and can also manifest as a mild glomerulonephritis and renal insufficiency (*2*). Eurasian hantaviruses (Hantaan virus, Puumala virus, and Seoul virus [SEOV]) cause HFRS; SEOV has a worldwide distribution (*1*). However, HPS is the clinical manifestation of most domestically acquired hantavirus infections in the United States. 

HFRS in the United States was first reported in 2008 in a 22-year-old man with SEOV infection (*2*). Investigating a SEOV outbreak in 2017, the Centers for Disease Control and Prevention described 17 human cases in 11 states, including Colorado (*3*). Of these 17 patients, 9 were asymptomatic, 8 became ill, and 3 were hospitalized and recovered. The index case-patient was an 18-year-old woman with hematuria and mildly elevated creatinine who owned a pet rat (*4*). Published descriptions of hantavirus infection with renal manifestations in the United States are few, and the clinical characteristics of the renal injury are not often described.

We report a 61-year-old man with predominant renal manifestations of hantavirus who acquired the virus in Colorado, USA, apparently after exposure to aerosolized rodent droppings. He lived on a farm in northeastern Colorado and had cleaned his garage of extensive mouse and rat droppings 2 weeks earlier. He did not wear a mask to clean and did not own pet rats or snakes. He sought care in April 2019 for a 4-day history of fever up to 38.3°C, headache, fatigue, and myalgia. He also reported abdominal pain, anorexia, neck stiffness, and photophobia. 

When he arrived he was alert; heart rate was 87 bpm, blood pressure 118/73 mm Hg, respiratory rate 26/min, temperature 37.0°C, and SpO_2_ 94%. Physical examination revealed mild abdominal tenderness. Laboratory findings included mild thrombocytopenia (140 × 10^9^/L; reference 150–100 × 10^9^/L); elevated creatinine (2mg/dL; reference 0.7–1.3 mg/dL); a glomerular filtration rate of 35 mL/min (reference 90–120 mL/min); and increased lactate dehydrogenase (306 units/L, reference 124–271 U/L), fractional excretion of sodium (FeNa) (0.5%), spot albumin-to-creatinine ratio (1,576 mg/G, reference <30 mg/G), and urine protein (100 mg/dL, reference 0–20 mg/dL). A 24-hour total protein excretion was 861 mg/D (reference 0–101 mg/D). Cerebrospinal fluid parameters were within reference values. Chest radiograph showed interstitial prominences and bibasilar patchy opacities (Figure). Renal ultrasound results were normal. Results were negative for blood and cerebrospinal fluid cultures and tests for HIV, hepatitis A/B/C, and lymphocytic choriomeningitis virus. He was admitted for overnight observation; intensive care was not required. Renal function was stable after 24 hours, and he was discharged home without renal replacement or other supportive therapy. 

**Figure:**
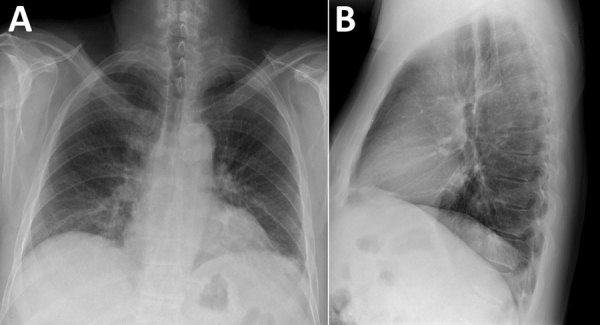
Chest radiographs displaying bibasilar patchy interstitial opacities in patient with hantavirus pulmonary syndrome, Colorado, USA. A) Posteroanterior view. B) Lateral view.

Using a qualitative assay (Quest Diagnostics Infectious Diseases, http://www.questdiagnostics.com), we detected IgM and IgG against recombinant hantavirus antigens derived from both Old and New World hantaviruses in patient samples. Testing at the Colorado Public Health Laboratory confirmed Sin Nombre virus (SNV)–specific IgM of 1:6,400 (expected result <1:100). The patient recovered by day 6, with no residual symptoms. Three weeks later, his creatinine was within reference levels (1.07 mg/dL).

This patient’s primary manifestations of hantavirus infection were reversible acute kidney injury and proteinuria. Respiratory symptoms were mild; his respiratory rate was slightly elevated, and chest radiograph disclosed interstitial infiltrates suggestive of mild HPS. Clinical HFRS manifestation can range from asymptomatic infection to acute renal failure with proteinuria, hematuria, and oliguria. Severity has been correlated with a specific hantavirus strain (*5*). Proteinuria is due to hantavirus nephritis, which is transient and usually resolves in 2 weeks (*6*). When acute interstitial nephritis occurs, it may manifest as acute tubular necrosis with histologic evidence of glomerular and endothelial damage (*7*).

HFRS caused by Puumala virus (nephropathia epidemica) or SEOV is generally mild and associated with a benign long-term prognosis. Diagnosis is facilitated by a high index of suspicion, a complete history including occupational and travel history, recognition of characteristic clinical symptoms, serologic or PCR testing, and exclusion of other infections. HFRS should be suspected in patients with acute renal failure, fever, hemorrhage, headache, and abdominal, back, or orbital pain; who live in rural areas; or who have possible rodent exposure within the previous 7 weeks. Because hantavirus infection rarely manifests as kidney disease in the United States, clinicians might be less aware of HFRS. At least 1 other SNV case has been reported with renal involvement requiring transient hemodialysis (*8*). General management is supportive, and most patients recover fully; few develop chronic renal sequelae (*9*).

Because of serologic cross-reactivity between SNV and SEOV, we lack a final etiology in this case. Our findings are also limited by the absence of viral sequencing and nephritis pathology analysis confirmation. However, our findings indicate that clinical recognition of renal signs and rapid detection of hantavirus infections can reduce risk for serious outcomes.
